# SHuffle, a novel *Escherichia coli* protein expression strain capable of correctly folding disulfide bonded proteins in its cytoplasm

**DOI:** 10.1186/1475-2859-11-56

**Published:** 2012-05-08

**Authors:** Julie Lobstein, Charlie A Emrich, Chris Jeans, Melinda Faulkner, Paul Riggs, Mehmet Berkmen

**Affiliations:** 1 New England Biolabs, Ipswich, MA 01938, USA; 2 Allopartis Biotechnologies, San Francisco, CA 94158, USA; 3 QB3-MacroLab, University of California, Berkeley, CA 94720, USA; 4 Bradley University, Peoria, IL 61625, USA; 5 New England Biolabs, 240 County road, Ipswich, MA 01938, USA

**Keywords:** SHuffle, Protein expression strain, Disulfide bond formation, Disulfide bond isomerization, DsbC, *trxB*, *gor*, ahpC*

## Abstract

**Background:**

Production of correctly disulfide bonded proteins to high yields remains a challenge. Recombinant protein expression in *Escherichia coli* is the popular choice, especially within the research community. While there is an ever growing demand for new expression strains, few strains are dedicated to post-translational modifications, such as disulfide bond formation. Thus, new protein expression strains must be engineered and the parameters involved in producing disulfide bonded proteins must be understood.

**Results:**

We have engineered a new *E. coli* protein expression strain named SHuffle, dedicated to producing correctly disulfide bonded active proteins to high yields within its cytoplasm. This strain is based on the *trxB gor* suppressor strain SMG96 where its cytoplasmic reductive pathways have been diminished, allowing for the formation of disulfide bonds in the cytoplasm. We have further engineered a major improvement by integrating into its chromosome a signal sequenceless disulfide bond isomerase, DsbC. We probed the redox state of DsbC in the oxidizing cytoplasm and evaluated its role in assisting the formation of correctly folded multi-disulfide bonded proteins. We optimized protein expression conditions, varying temperature, induction conditions, strain background and the co-expression of various helper proteins. We found that temperature has the biggest impact on improving yields and that the *E. coli* B strain background of this strain was superior to the K12 version. We also discovered that auto-expression of substrate target proteins using this strain resulted in higher yields of active pure protein. Finally, we found that co-expression of mutant thioredoxins and PDI homologs improved yields of various substrate proteins.

**Conclusions:**

This work is the first extensive characterization of the *trxB gor* suppressor strain. The results presented should help researchers design the appropriate protein expression conditions using SHuffle strains.

## Background

Many research applications require the purification of high yields of an active and correctly folded protein for either its study (biochemical analysis, X-ray crystallography, etc.), or for its direct use (e.g. as in therapeutic and diagnostic applications). In general, protein overexpression, and the generation of high yields is oftentimes difficult and unpredictable. It becomes even more arduous when the protein of interest contains post-translational modifications, such as disulfide bonds, which are critical for proper protein folding, stability, and/or activity. Disulfide bonds are formed by the oxidation of sulfhydryl groups between two cysteine side chains resulting in a covalent bond, greatly increasing the stability of a protein. A large proportion of proteins contain disulfide bonds. For example, analysis of the human genome revealed that 30% of the proteins are predicted to be targeted to the endoplasmic reticulum (ER) where disulfide bond formation is compartmentalized and of those, half are predicted to form disulfide bonds [1]. Since disulfide bonds increase the stability of proteins, most disulfide-bonded proteins are secreted or remain anchored to the plasma membrane, exposed to the environment. This feature of disulfide-bonded proteins makes them excellent therapeutic agents or targets for the pharmaceutical industry. Recent market analysis of therapeutic proteins indicates that all classes of therapeutic proteins are composed mostly or exclusively of proteins containing disulfide bonds [2]. It is therefore critical to have multiple expression systems which can express disulfide-bonded proteins rapidly with relative ease and low cost. Additional molecular tools must also be developed to fine tune the protein expression conditions for a given substrate protein, to achieve maximal yields to high purity.

Currently there are several expression systems available for the production of disulfide-bonded proteins, with each system having its own advantages and disadvantages. Although eukaryotic expression systems such as Chinese Hamster Ovary (CHO), yeast or insect cells offer the capacity to express complex multi-disulfide-bonded proteins, these systems are slow and expensive. Cell-free expression systems may have circumvented the problem of speed but are not feasible for scale-up. For most applications, prokaryotic expression remains the most attractive expression system due to its relatively low cost, high speed, ease of use, high yields, and the availability of large numbers of genetic tools for optimization purposes.

*Escherichia coli* is the most popular choice for recombinant protein production. Currently there are only a handful of *E. coli* expression strains commercially available. There is an ever growing demand for new, versatile and improved protein expression strains, especially those that are engineered to handle post-translational modifications such as disulfide bond formation. So far, production of soluble and active disulfide-bonded proteins to high yields in *E. coli* remains a challenge. This is mainly due to the fact that for most overexpression systems, the recombinant protein produced is expressed in the cytoplasm, but disulfide bond formation is compartmentalized to the periplasm where *E. coli* is poorly adapted for producing multi-disulfide bonded proteins in high yields. Since all living cells studied to date have enzymes dedicated to reducing disulfide bonds in their cytoplasm, the formation of disulfide bonds have been compartmentalized to extra-cytoplasmic compartments such as the periplasm in gram negative bacteria [3] or the ER in eukaryotes [4]. Thus, proteins which require disulfide bonds for their folding and stability are poorly expressed, misfolded, and are not active when expressed in the cytoplasm of *E. coli*.

A major breakthrough came through the pioneering work conducted by Beckwith and co-workers during their studies into the redox pathways of *E. coli*[5-8]. The culmination of their work along with several other labs elucidated the cytoplasmic redox pathways and enzymes in *E. coli.* This knowledge enabled the Beckwith lab to engineer a mutant *E. coli* strain capable of promoting disulfide bond formation in the cytoplasm [9].

The formation of a disulfide bond is catalyzed by enzymes belonging to the thioredoxin super-family [10]. In *E.coli*, disulfide bond formation is catalyzed in the periplasmic space by the enzyme DsbA [3]. DsbA is one of the strongest oxidases measured and will oxidize cysteine residues consecutively as they enter the periplasm [11,12]. Proteins which require multiple non-consecutive disulfide bonds require the action of a disulfide bond isomerase to shuffle the disulfide bonds within the mis-oxidized protein to produce its native folded state [13,14]. *E. coli’*s periplasmic disulfide bond isomerase is DsbC, a homodimeric “V” shaped protein, where each arm of the “V” is a thioredoxin fold brought together by a dimerization domain [15]. The cleft formed by the V-shaped DsbC is hydrophobic, thought to preferentially interact with mis-oxidized proteins that have their core hydrophobic residues exposed. This hydrophobic cleft is also hypothesized to mediate the chaperone property of DsbC, which is independent of its redox active cysteines [16]. Over-expression of DsbC greatly enhances the amount of correctly folded protein in vivo both in the periplasm [17,18] and in the cytoplasm [8,19,20]. Incubation of DsbC in vitro in cell free expression systems has also been shown to enhance the amounts of correctly folded disulfide bonded proteins [21,22].

The engineering of an *E coli* strain to produce large quantities of cytoplasmic protein with disulfide bonds would require engineering of the two reductive pathways (thioredoxin and glutaredoxin/glutathione) in the cytoplasm. Due to the presence of numerous thiol reductases (Grx1, Grx2, Grx3, Trx1, Trx2), glutathione, and small thiol reductants, cysteines are maintained in their reduced state in the cytoplasm of wild type *E. coli* and are not able to form stable disulfide bonds (they may still form transiently [23-25]). To genetically engineer a strain that allows the formation of stable disulfide bonded proteins within the cytoplasm, thioredoxin reductase (*trxB*) and glutathione reductase (*gor*) were mutated. Mutant *E. coli* cells carrying deletions of *trxB gor* are nonviable as certain essential proteins, such as ribonucleotide reductase, cannot be re-cycled back to their active reduced states [26]. A suppressor screen for *trxB gor* lethality generated a strain (FÅ113) whose mutant peroxidase AhpC* had gained the ability to reduce Grx1, restoring reducing power to the cell [7]. Thioredoxins remain in their oxidized state and can oxidize protein substrates which require disulfide bonds for their folding [6]. This mutant *E. coli* strain (FÅ113) is sold commercially under the name Origami by Novagen. However, in this strain, thioredoxins, like DsbA, form disulfide bonds indiscriminately, resulting in some proteins being mis-oxidized and inactive. A marked increase in activity of some cytoplasmically expressed proteins was observed when DsbC lacking its signal sequence was co-expressed in the cytoplasm [8,9,27]. Recently, co-expression of the yeast sulfhydryl oxidase Erv1p has also been shown to improve production of disulfide bonded proteins in the cytoplasm of *E. coli*[28,29]. Even though this work demonstrates the various methods of producing disulfide bonded proteins, expression of cytoplasmic DsbC was still crucial in achieving high yields of correctly folded substrate protein. While this method is in its infancy, utility of this system has already been demonstrated [30].

The *E. coli trxB gor* suppressor has been a useful strain for producing disulfide bonded proteins resulting in hundreds of publications since the utility of this strain was first shown in 1999 [8]. However, no comprehensive study has been conducted on the parameters involved in producing correctly folded protein within this strain. Furthermore, although the co-expression of cytoplasmic DsbC had been shown to improve protein folding [20], no such strain was engineered nor studied in detail. Here, we present a novel protein expression strain based on a different *trxB gor* suppressor strain (SMG96). We engineered this strain to cytoplasmically over-express DsbC under the relatively strong and highly-regulated rRNA promoter *rrnB*[31]. We characterized the redox state of the strain and investigated the effects of varying three common parameters (temperature, time and strength of induction) on protein expression. Using the optimized conditions, we expressed and purified eight different substrate proteins and showed their relative solubility. Finally, we co-expressed a set of helper proteins and evaluated their ability to increase the folding of a subset of proteins. This strain is currently commercially available under the name SHuffle from New England Biolabs.

## Results

### Redox state of SHuffle cells are altered to permit oxidative folding

**Figure 1:**
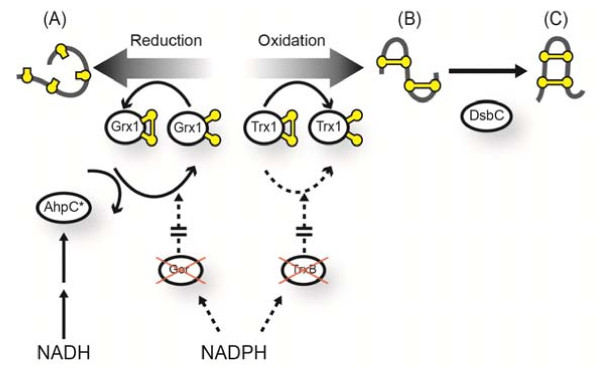
**Disulfide bond formation in the cytoplasm of SHuffle.** Schematic diagram of the redox pathways in the cytoplasm of SHuffle. Dotted lines represent disabled protein interactions due to the deletion of *trxB* and *gor*. Redox state of cysteines (yellow balls) are indicated (oxidized = ball + stick; reduced = ball). **(A)** Protein is reduced by Grx1 or oxidized by Trx1. **(B)** Mis-oxidized protein is isomerized to its native correctly folded state **(C)** by DsbC.

We constructed a mutant *E. coli* strain with an altered redox state that permits the formation of stable disulfide bonds within its cytoplasm. This strain’s parent is the previously described *E. coli* strain SMG96 [32] which itself is based on the strain FÅ113 [8]. SMG96 lacks the *gor* and *trxB* reductases; the lethality conferred by these mutations is suppressed by a mutation in the peroxidase ahpC* [7]. Figure 1 shows a schematic of this altered redox pathway which results in the reduction of Grx1 by AhpC*, restoring viability. Trx1 remains oxidized and therefore catalyzes the formation of disulfide bonds within the cytoplasm (Figure 1B). We have further engineered the strain to express DsbC in the cytoplasm, which should isomerize mis-oxidized proteins to their native states (Figure 1C).

### Expression cytoplasmic DsbC in SHuffle can improve oxidative folding

**Figure 2:**
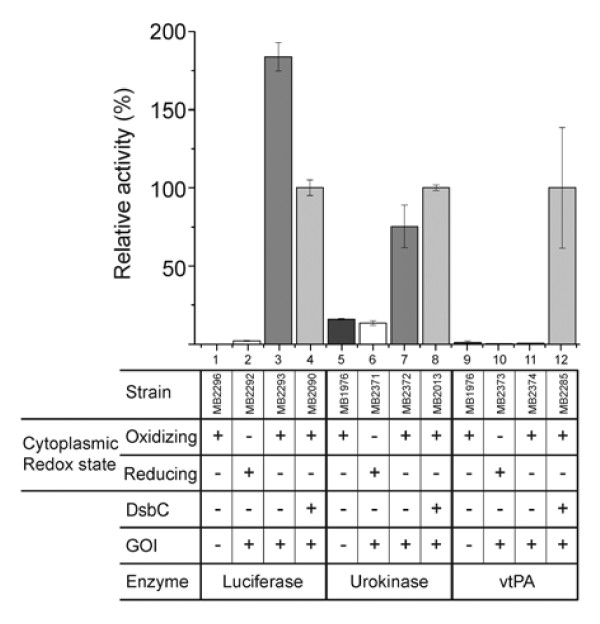
**Influence of cytoplasmic DsbC on three different proteins in SHuffle.** Relative enzymatic activities of various proteins (luciferase, urokinase, vtPA) measured from crude lysates. Cytoplasmic redox state, presence of cytoplasmic DsbC and gene of interest (GOI) are indicated.

DsbC is an oxido-reductase chaperone, capable of enhancing the oxidative folding of proteins both in its native periplasmic compartment and when expressed cytoplasmically [8,19,20]. To investigate the role of cytoplasmic DsbC in SHuffle cells, we compared the activity of three different proteins which require disulfide bonds to achieve their native folded state (Figure 2). *Gaussia luciferase* has 10 cysteines which are all involved in disulfide bonds, although the pattern of disulfide bonds remains unknown [33]. As schematically depicted in Table 1, urokinase and vtPA both have non-consecutive disulfide bonds with 18 and 12 cysteines, respectively, making them ideal candidates for testing the role of cytoplasmic DsbC.

**Table 1:** Table summarizing optimum expression conditions for all proteins produced in SHuffle

**Substrate**	**#cysteine**	Disulfide bond connectivity*	**Promoter**	**Optimum conditions**			**Yield mg/l**	**Specific Activity U/mg**
				**T °C**	**[IPTG] mM**	**Time of induction**		
vtPA N-HIS	12		T7	16	1.00	Mid log	0.2	721
				16	Auto	Autoexpression	1.2	159
Gluc	10		T7	37	1.00	Mid log	13.8	8.1E + 10
AppA	8		T7	37	0.01	Late log	51.3	6.74
Cel9A	6		T7	25	Auto	Autoexpression	250	320.4
PhoA	4		T7	25	0.05	Mid log	40.2	5.6E + 06
Chitinase	3		T7	16	0.10	Late log	7.1	4.5E + 09
CelZ	2		T7	25	Auto	Autoexpression	400	164.4

* Solid lines represent known disulfide bonds from crystal structures, dotted lines represent cysteines predicted to be involved in disulfide bonds with unknown structures.

We measured the activities of the three candidate enzymes in four different strain backgrounds to determine what effects an oxidizing cytoplasm and the presence of DsbC in the cytoplasm have on their activity. As expected, no or very little enzyme activity was detected in cell lysates lacking the gene of interest (GOI) (Figure 2 lane 1, 5 and 9). A similar lack of enzyme activity was observed in wild type *E. coli*, suggesting that the proteins do not fold correctly in a normal reducing cytoplasm (Figure 2, lane 2, 6 and 10). In contrast, when we expressed the enzymes in an oxidizing cytoplasm, we observed a marked increase in activity for luciferase and urokinase but not for vtPA (Figure 2, lane 3 and 7), suggesting that an oxidizing cytoplasm is sufficient for the correct folding of only some proteins that contain disulfide bonds (Figure 2, lane 11). Cytoplasmic DsbC increased the activity for two of the three candidates. Urokinase activity slightly increased in the presence of DsbC (Figure 2, lane 8), whereas vtPA was completely dependent on DsbC for proper folding (Figure 2, lane 12). Luciferase activity was reduced almost two-fold in the presence of DsbC (Figure 2, lane 4). These results suggest that DsbC can be absolutely essential for folding of certain protein substrates. We suggest that SHuffle is an important strain background for researchers to use when expressing disulfide-bonded proteins that display low activity in other strain backgrounds. Furthermore, we conclude that SHuffle’s effect on the folding of disulfide-bonded proteins is substrate protein specific.

### Expression of proteins in SHuffle B strains results in greater yields compared to SHuffle K12 strains

**Figure 3:**
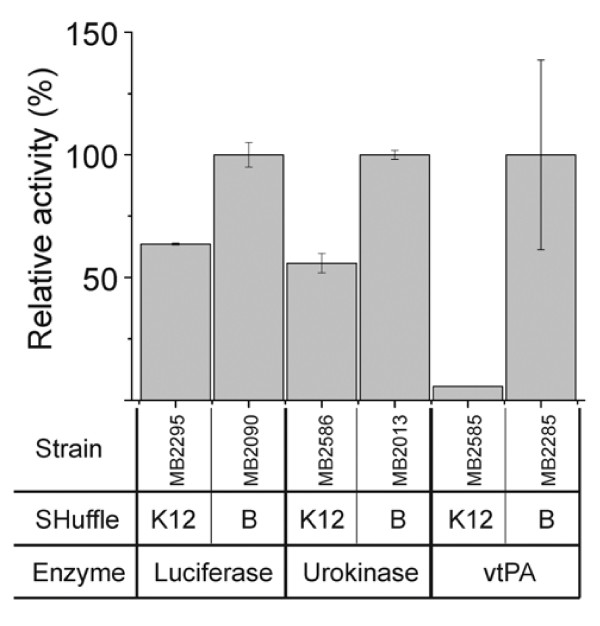
**Comparison of relative enzymatic activities in SHuffle (*E. coli *K12 : C3025 or C3026) and SHuffle express (*E. coli *B : C3028 or C3029).** Relative enzymatic activities of various proteins (luciferase, urokinase, vtPA) measured from crude lysates of various SHuffle strains in the K12 or B strain backgrounds.

During the course of our experiments, we noticed differences in the activities of proteins measured from SHuffle cells constructed in the K12 vs. the B strain backgrounds. In order to determine that the differences were not due to growth rate, we measured growth of cultures at 30ºC. We observed no significant difference in growth rate between SHuffle cells and their parental wild type (Additional file 1). To directly compare the effect of strain background, we measured the activities of three different substrate proteins expressed in either SHuffle K12 (C3025 or C3026) or SHuffle B (C3028 or C3029) (Figure 3). Luciferase and urokinase activities were approximately 2-fold higher in the B background than in K12. Expression of vtPA did not result in any detectable activity when produced in the K12 background, but was active in the B background. We confirmed our observation with western blot analysis and detected vtPA only in SHuffle B strains and not in SHuffle K12 (Supplementary material Figure 2). Thus, in the case of all three substrate proteins, we observed consistently higher enzyme activities in SHuffle B strains compared to K12.

We wished to explore whether the observed differences were due to differences in the mechanism of suppression of *trxB gor* lethality. Therefore, we sequenced the *ahpC * gene in SHuffle K12, SHuffle B, their parental wild type strains, and 16 new suppressors isolated using the method described previously [32]. While SHuffle K12 contained the previously described triplet codon expansion ahpC* allele [7], 15 out of the 16 newly isolated SHuffle B strains had a novel triplet codon contraction allele (*ahpC*^Δ^) and only one isolate had the classic triplet codon expansion (Table 2). We did not observe any significant difference in vtPA activity in SHuffle B ahpC*  versus *ahpC *^Δ^ cells (data not shown). Even though the mechanism of disulfide bond formation did not appear to vary between the two suppressors, *E. coli * K12 and B might have distinct cellular responses to oxidative stress. To test this hypothesis, we grew cells in microtiter dishes with varying amounts of hydrogen peroxide. *E. coli * B cells ceased to grow at concentrations above 4 mM hydrogen peroxide, while *E. coli * K12 strains ceased to grow above 10 mM hydrogen peroxide (data not shown). We also compared the hydrogen peroxide sensitivity of SHuffle B cells having either ahpC* or *ahpC *^Δ^ suppressors mutations. Both strains displayed similar levels of sensitivity and ceased to grow at hydrogen peroxide concentrations above 6 mM (data not shown). Thus, we conclude that the differences in enzyme activities observed for K12 and B strains (Figure 3) are not due to the nature of the suppressing mutation in the two strain backgrounds but instead are more likely to be due to general genetic differences between the two strains.

**Table 2:** **Genomic sequence of *ahpC *in various SHuffle strains**

**Strain**	**amino acid #**	**34**	**35**	**36**		**37**	**38**	**39**	**40**
	**amino acid**	**Ser**	**Val**	**Phe**		**Phe**	**Phe**	**Tyr**	**Pro**
wt *E. coli * K12 and B	*ahpC*	AGC	GTC	TTC		TTC	TTC	TAC	CCG
SHuffle K12	ahpC*	AGC	GTC	TTC	TTC	TTC	TTC	TAC	CCG
SHuffle B	*ahpC*^Δ^	AGC	GTC	TTC			TTC	TAC	CCG

### Cytoplasmic DsbC in SHuffle cells are in their active hemi-reduced state

The redox state of DsbC is critical for its isomerase/reductase activity both in vivo [34] and in vitro [35]. In order to function as a disulfide bond isomerase, DsbC must be maintained in its hemi-reduced state. Each DsbC monomer contains 4 cysteine residues. The N-terminal redox active cysteines (Cys98–Cys101) face the hydrophobic cleft and are maintained in a reduced form in the periplasm by the inner membrane protein DsbD [34]. The C-terminal pair (Cys140-Cys163) form a stable disulfide bond that is critical for the folding and stability of DsbC [36]. In the absence of DsbD, DsbC becomes oxidized and cannot function as an isomerase/reductase and instead can now function as an oxidase [37]. Unlike the periplasm, the cytoplasm lacks a dedicated reductase such as DsbD to maintain the active site cysteines of DsbC in its reduced state. Furthermore, the reducing/oxidizing conditions of the cytoplasm of SHuffle cells may not be able to maintain cytoplasmic DsbC in its hemi-reduced state. It is therefore critical to understand the exact redox state of cytoplasmic DsbC in SHuffle cells.

**Figure 4:**
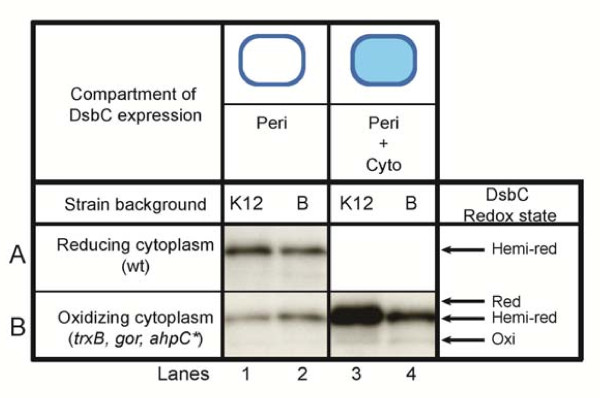
**Redox state of DsbC.** Redox state of AMS alkylated DsbC analyzed by western blot using anti-DsbC antibody. Redox states of DsbC are indicated as either hemi-reduced (hemi-red), reduced (red) or oxidized (oxi). (**A**) Redox state of periplasmic DsbC in wild type *E. coli* K12 (MB10; lane 1) and in *E. coli* B (C2566; lane 2), (**B**) Redox state of DsbC in the oxidizing E. coli ∆trxB, ∆gor, ahpC*, in the periplasm of *E. coli* K12 (MB932; lane 1), in *E. coli* B (MB1731; lane 2) and when expressed both in the periplasm and cytoplasm of SHuffle K12 (C3026; lane 3) or SHuffle B (C3029; lane 3).

We investigated the redox state of DsbC using AMS alkylation followed by western blot analysis using anti-DsbC antibody (Figure 4). AMS alkylates any free thiol group found in the side chains of cysteine residues, covalently adding 500 Daltons, resulting in mobility shift in SDS-PAGE analysis. Since SHuffle cells contain both periplasmic and cytoplasmic copies of DsbC, we first investigated the redox state of periplasmic DsbC in the parent strain of SHuffle K12 and SHuffle B. In both wild type *E. coli* K12 and B strains, periplasmic DsbC was detected mostly in its active hemi-reduced state at similar levels of expression (Figure 4A, lane 1 and 2). Similar amounts of periplasmic DsbC were detected in K12 and B strains which had the trxBgorahpC* mutations (Figure 4B, lane 1 and 2). Significantly higher amount of hemi-reduced DsbC was detected in SHuffle K12 cells, indicating that cytoplasmic DsbC is overexpressed from the chromosome and is in the correct redox state to function as a disulfide bond isomerase (Figure 4B, lane 3). However, SHuffle B cells did not over-express cytoplasmic DsbC to the same level as SHuffle K12 cells (Figure 4B, lane 4). This may have to do with differential regulation of the *rrnB* promoter in *E. coli* B cells in comparison to *E. coli* K12, since the *rrnB* promoter controls the expression of cytoplasmic DsbC. In order to understand whether cytoplasmic DsbC is under-expressed and limited in SHuffle B cells, we constructed two more SHuffle B cells in which the DsbC was under the regulation of rrnB promoters with 9 or 70 times higher transcriptional activity [31]. These strains did not show any improvement in the activity of urokinase when compared to SHuffle B, suggesting that cytoplasmic DsbC is sufficiently over-expressed (data not shown).

The culmination of these results when combined with the in vivo protein expression data indicates that the majority of cytoplasmic DsbC is active in its hemi-reduced state, essential for its disulfide bond isomerase activity. We also observed significant amounts of oxidized cytoplasmic DsbC in SHuffle cells, which may directly contribute to the oxidation of substrate proteins.

### Optimization of protein expression conditions

To optimize production of proteins in SHuffle cells, we investigated the effects of three parameters on the expression of seven different substrate proteins. In consideration of the average researcher who expresses proteins using a shake flask system with limited time and resources, we chose the three most commonly modified parameters: temperature, time of induction, and concentration of inducer (IPTG).

#### Temperature

The effect of temperature on protein folding has been well documented and is one of the most common factors to be optimized during production of proteins [38]. We therefore investigated the role of temperature on protein expression by growing SHuffle cells in rich medium initially at 30˚ until the cells reached mid log growth phase. Protein expression was induced with 1 mM IPTG and the growth temperature shifted to 16°C, 25°C, 30°C or 37°C. At the end of exponential growth, activity of the substrate protein was measured. As shown in Table 1, the optimal temperature varied among the seven proteins: for two it was 16°C, for three it was 25°C, and for the final two it was 37°C. We conclude that the effect of temperature was protein specific.

#### Time of induction

Using the optimal temperature discovered in the prior experiment, we investigated the effect of inducing at various growth phases. SHuffle cells were grown at the optimal temperature and were induced with 1 mM IPTG at the initial time of inoculation, mid-log or late-log growth phase. Further downstream processes were the same as described above. In the case of the two cellulases (CelZ and Cel9A) an additional method of induction, termed here ‘autoexpression’ was tried and found to be optimal over standard IPTG induction (Table 1). Autoexpression relies on the diauxic response of *E. coli* when grown in multiple carbon sources such as glucose and lactose, resulting in induction of *lac* promoter upon depletion of glucose [39]. Using Magic Media supplied by Invitrogen, cells were grown overnight without induction and enzymatic assays were performed the next day. Further characterization of autoexpression was performed by comparing the yields obtained for a poor folding protein such as vtPA, when expressed under optimized IPTG conditions vs. autoexpression. The yields of purified vtPA increased from marginally detectable amounts to over 1 mg/l, indicating that autoexpression may be a suitable method of protein production in SHuffle cells (Table 1).

#### Concentration of inducer

Using the optimal expression temperature and time of induction conditions discovered prior, the concentration of inducer was optimized. SHuffle cells were grown at the optimal temperature and were induced with various concentrations of IPTG (0, 0.01, 0.05, 0.1 and 1 mM) at the optimal growth phase of induction. The optimal concentration of inducer was protein-specific, varying from 0.01 mM to 1 mM (Table 1).

**Figure 5:**
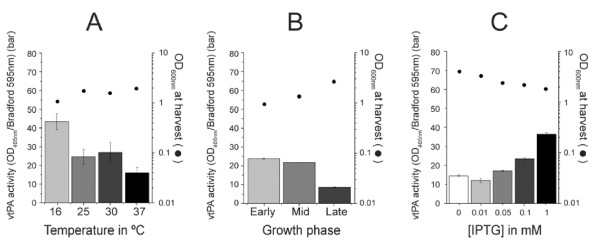
**Optimizations of vtPA expression in SHuffle express T7 grown in shake flask.** Activity of vtPA from crude lysates of C3029, (**A**) grown at various temperatures, (**B**) induced at various growth phases, and (**C**) with various concentrations of IPTG. The activity of vtPA is indicated on the left Y-axis (bar). OD_600nm_ measured at time of cell harvest is indicated on the right Y-axis (filled circle).

An example of this optimization process is shown for vtPA (Figure 5). Using our optimization process, the optimum shake flask expression condition for vtPA was growth at 16°C during protein expression, with 1 mM IPTG induction at mid-growth phase. Overall, our results indicate that the optimal conditions for protein expression in SHuffle cells are protein-specific. However, we did note that temperature had the most profound effect and lowering of growth temperature during induction usually resulted in improved yields. While we did not investigate autoexpression systematically with all the proteins, this induction method also gave improved yields where it was used. Thus, a thorough study is required to optimize the expression conditions for any given new protein of interest.

### Proteins expressed in SHuffle cells results in diverse levels of solubility

The solubility of a protein is an important indicator of its correct folding as determined by functional binding [40] or enzymatic [41] assays. Determining a protein’s solubility will help guide the researcher design the correct experimental procedure to improve its yield. For example, a protein having only 5% of the total expressed protein soluble will require optimization of its folding pathway while another protein having 90% solubility might require increased expression levels to improve yields. We therefore quantified the levels of solubility of each of the proteins we expressed to assess the level of success of folding in SHuffle strains.

**Figure 6:**
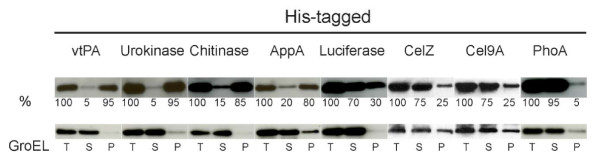
**Solubility of proteins expressed with optimal conditions in SHuffle.** Cells were lysed and total cells lysates (**T**) were separated into soluble (**S**) and pellet (**P**) fractions. Samples were analyzed using western blot with the appropriate antibody and percentages of solubility are indicated. Anti-GroEL antibody was used as loading control.

Using the panel of seven substrate proteins expressed under the optimum conditions we discovered previously, cell lysates were produced as described in methods. An aliquot of each lysate was removed to represent the total amount of protein (T). Samples were subjected to centrifugation with the supernatant representing the soluble fraction (S) and the pellet representing the insoluble fraction (P). Samples were analyzed by western blot with the appropriate antibody. As a control for proper fractionation, samples were also probed with anti-GroEL antibody to detect the soluble fraction that contains GroEL. As expected, protein solubility varied a great deal. Solubility ranged from 5% for poorly folding substrate proteins such as vtPA and urokinase to 95% for protein substrates that fold efficiently such as PhoA (Figure 6). These data highlight the fact that the solubility of a protein is highly dependent on the nature of the protein and high levels of soluble protein can be achieved when over-expressed in SHuffle cells.

### Co-expression of helper proteins can improve oxidative folding

Folding of disulfide bonded eukaryotic proteins in a prokaryotic host is challenging. For any given protein, there may be one or more bottlenecks in its folding pathway that occur when the folding of the protein is decoupled from its native host environment. Reasons for inefficient folding are diverse and unique for each protein and may be due to: the lack of intrinsic folding properties of the protein (e.g. rate of translation governed by codon usage), the physical environment (e.g. folding in a specialized compartment) or the dependence on a set of chaperones dedicated to the folding of the nascent polypeptide in the native host. This problem is highlighted by the variation in the solubility of the proteins we expressed in SHuffle. To increase the capacity of SHuffle cells to fold a greater variety of disulfide bonded proteins, we co-expressed numerous “helper” proteins based on our assumption that they may alleviate a folding bottleneck that may exist for a given protein. We therefore chose our least soluble proteins (vtPA, urokinase and chitinase) as indicators of folding improvement, as we hypothesized that these proteins would allow the largest range of improvement. To facilitate improved folding of these proteins, we co-expressed 16 different helper proteins which could subdivided into three general categories: redox active, chaperone and oxidative stress. All of the helper genes were cloned into pBAD34 expression vector with a pACYC origin of replication, under the regulation of the arabinose promoter. A second set of C-terminally flag-tagged constructs were constructed in order to assess the expression levels of the helper proteins using western blots probed with anti-flag antibodies. Full length proteins were detected for all of the helper proteins except PDI, which could be detected upon longer exposure (Supplementary material Figure 3). SHuffle cells expressing vtPA, urokinase or chitinase along with one of the helper plasmids were grown under the optimal expression conditions discovered prior. Expression of the helper protein was induced in the beginning of growth by adding final concentration of 0.2% (w/v) L-arabinose and the substrate protein was induced once the cells reached mid log growth phase. Enzymatic activities were measured and normalized to cells expressing vector alone (pBAD33). The results are summarized in Table 3. Overall, we found that co-expression of helper proteins dramatically improved the yield of vtPA (up to 11-fold) while only slightly improving the yields of urokinase and chitinase (less than 2-fold for the best helper). An in-depth description of these results is below.

**Table 3:** Effects of co-expression of helper proteins on substrate protein activity

	**Helper**	**vtPA**	**Urokinase**	**Chitinase**
	pBAD33	1.00	1.00	1.00
				
	cAaDsbC	5.92	1.17	0.32
	cDsbC	0.19	1.19	0.97
	TrxA_CGPC_	4.50	1.16	1.19
	TrxA_CPYC_	**9.83**	1.05	0.85
	TrxA_CPHC_	**7.73**	1.02	0.71
Redox active Helpers	cAaPDO	3.31	0.58	0.76
	QSOX	**8.12**	0.86	1.02
	PDI	3.08	**1.52**	**(1.58)**
	EUG1	5.43	**1.31**	**1.24**
	MPD1	4.88	1.10	**1.42**
	MPD2	**8.91**	**1.37**	1.18
Chaperone helpers	MalE	0.87	1.11	1.13
	HlpA (skp)	6.97	1.16	0.53
	KatG	**(11.81)**	0.90	1.05
Oxidative stress helpers	AhpC, AhpF	5.69	**(1.84)**	0.90
	AhpC*, AhpF	4.29	**1.30**	1.08

Brackets indicates the best fold improvements while bold are the following best fold improvements.

#### Redox active helpers

It is possible that the mechanism of disulfide bond formation in the cytoplasm of SHuffle cells is not optimal for the correct folding of a given protein. There may not be sufficient disulfide bond isomerase (DsbC) for the abundance of overexpressed substrate proteins. To assess this, we expressed DsbC lacking its native signal peptide. No significant improvement in activity of urokinase and chitinase were detected upon increased levels cytoplasmic DsbC (Table 3), indicating that sufficient amounts of DsbC are expressed in SHuffle cells and that disulfide bond isomerization is not the folding bottleneck for these proteins. However, vtPA activity was reduced ~5-fold in SHuffle strains in comparison to isogenic strains lacking cytoplasmic DsbC (Table 3).

The role of thioredoxins in the formation of disulfide bonds within the *trxB* suppressor strains has already been demonstrated [6]. Furthermore, co-expressing mutant thioredoxins with altered active sites has resulted in significant improvement in protein production [8]. We therefore chose the two mutant thioredoxins with altered active sites along with the wild type (CGPC = wt, CPYC = Grx1, CPHC = DsbA) to assess whether co-expressing thioredoxins could assist in the formation of correctly oxidized substrates. Co-expression of thioredoxins increased the activity of vtPA up to 10-fold but did not result in any improvement in the case of urokinase and chitinase.

Protein disulfide isomerase (PDI) is an essential ER resident oxido-reductase involved in the oxidation and isomerization of disulfide bonded proteins in eukaryotes. In vitro it catalyzes the oxidative formation, reduction, or isomerization of disulfide bonds depending on the redox potential of the environment [42]. Expression of PDI in *E. coli* has already been demonstrated with mixed success. Co-expression of yeast PDI in the periplasm resulted in a 50% increase in the yield of tissue plasminogen activator (tPA), while rat PDI had no beneficial effect both in the periplasm and cytoplasm [43]. Due to this apparent substrate specificity of PDI’s, we decided to co-express various PDI homologs from *Saccharomyces cerevisiae* (PDI, EUG1, MPD1 and MPD2). Co-expression of the PDI homologs was the most successful class of helper proteins. In the case of urokinase and chitinase, PDI homologs were the best helper proteins while in the case of vtPA only one PDI homolog (MPD2) was second best helper protein (Table 3).

Sulfhydryl oxidases, such as human quiescin-sulfhydryl oxidase (QSOX) [44], can catalyze the formation of disulfide bonds through their FAD cofactor, resulting in the reduction of oxygen to hydrogen peroxide [45]. We chose QSOX as a helper protein, as co-expression of other sulfhydryl oxidases enhances production of disulfide bonded proteins in the cytoplasm of *E. coli*[28-30]. Although co-expression of QSOX increased vtPA activity 8-fold, it had no positive influence on the expression of urokinase and chitinase (Table 3).

Another candidate as a helper protein was the archeal cytoplasmic protein disulfide oxidoreductase (PDO) which can catalyze disulfide bond formation in vitro [46,47]. We chose the PDO from *Aquifex aeolicus*, as this species has been predicted to have one of the most oxidizing cytoplasms [48]. Co-expression of the *A. aeolicus* VF5 PDO did not result in any significant improvement in the yields of vtPA, urokinase or chitinase (Table 3).

#### Chaperone helpers

As a fusion protein, maltose binding protein (MBP) promotes folding and increases the solubility of its fused cargo [49]. We co-expressed MBP as a helper protein but did not observe any significant improvement in the yields of vtPA, urokinase and chitinase. This may be due to the observation that MBP is most successful at increasing solubility when fused N-terminally [50], indicating that MBP may need to act on the elongating polypeptide and may not act as a chaperone post-translationally when not fused. Another periplasmic chaperone we expressed within the cytoplasm of SHuffle was the “seventeen kilo Dalton protein” (Skp) known to have a broad range of interacting substrates [51]. Cytoplasmic co-expression of Skp improves the folding of certain eukaryotic proteins [52]. However, no positive effects on folding of our test proteins were observed when Skp was co-expressed (Table 3).

#### Oxidative stress helpers

SHuffle cells lack *trxB* and *gor* and cannot efficiently reduce oxidized proteins. This result in the buildup of oxidized inactive proteins, which induces a general oxidative stress response, mediated by the transcriptional factors OxyR and the SoxRS regulon [53,54]. In addition, AhpC* has lost its function as a peroxidase resulting in the accumulation of hydrogen peroxide. This can cause oxidative damage to proteins and may diminish cell viability, which in turn, may lower the yield of recombinant protein production. Under such conditions, the expression of the catalase gene *katG*, which scavenges and removes hydrogen peroxide and the peroxidase AhpC is highly up regulated [55]. However, native defense mechanisms may not be sufficient, as SHuffle cells have three of its reductive pathways disrupted (glutathione, thioredoxin and peroxiredoxin pathways). We therefore chose KatG and AhpCF and the peroxidase deficient mutant AhpC*F as candidate helper proteins to combat oxidative stress. Expression of *katG* resulted in 12-fold increase in the activity of vtPA, making it the best helper protein, while expression of AhpCF and AhpC*F had modest effects on vtPA. In the case of urokinase, co-expression of either AhpCF or AhpC*F resulted in the best improvements in activity. In the case of chitinase, none of these helpers had any effect (Table 3). Taken together, these results further highlight the protein specific nature of protein folding and the lack of predictability in deciding which molecular chaperone system will improve protein solubility [56].

## Discussion

In this manuscript we present a novel *E. coli* strain based on the *trxB gor* suppressor strain SMG96. We further engineered into its chromosome a *dsbC* gene lacking its signal sequence, under the regulation of the strong ribosomal promoter *rrnB*. These strains were engineered both in *E.coli* K12 and B strain backgrounds. A detailed characterization of the SHuffle strains along with parameters involved in protein production at bench-scale (non-high throughput) was investigated.

To expand our understanding of the mechanism of disulfide bond formation within SHuffle strains, we investigated the redox state of cytoplasmic DsbC. We showed that the majority of cytoplasmic DsbC is in its hemi-reduced state, which is essential for its disulfide bond isomerase activity. However, oxidized DsbC species were also detected when expressed within the oxidizing cytoplasm, which could result in DsbC directly oxidizing reduced substrates. This is not surprising, as mutant DsbB which have gained the ability to oxidize DsbC are in turn capable of oxidizing proteins in the periplasm [57]. Oxidized DsbC species may not always be beneficial to the folding of reduced proteins which require disulfide bonds. This may explain the drop in activity observed for *Gaussia* luciferase when expressed in cells with cytoplasmic DsbC. Similar observations were made when expressing parathyroid hormone in *trxB gor* strains [58]. In this study, co-expression of cytoplasmic DsbC had no positive influence in vivo, but did dramatically reduce the amount of misfolded species when DsbC was co-incubated in the presence of oxidized and reduced glutathione.

*E. coli* B strains such as BL21 are the preferred host strain for protein expression as generally give higher yields for the large majority of proteins. Some of the reasons for this may be that, unlike its K12 cousin, it has not been subjected to extensive domestication for the purpose of DNA manipulation [59], and it lacks the cytoplasmic protease *lon* known to play a key role in protein quality control [60]. Similarly, when we compared the expression of three proteins in SHuffle K12 vs. SHuffle B strains, we consistently observed higher yields in the B strain backgrounds. However, we also observed differences between the two strains at the level of redox states of proteins. Unlike in SHuffle K12, a fraction of periplasmic DsbC was observed to be in its reduced state in the SHuffle B strain. Further redox differences were observed in the *ahpC* mutations between the two strains. While SHuffle K12 *ahpC* gene has the triplet TTC codon expansion, SHuffle B *ahpC* gene has the triplet codon contraction, lacking one of the three TTC codons. These differences highlight the distinct biological differences between the two SHuffle strains and require detailed studies to elucidate their biological roles.

To define conditions critical for the folding and correct formation of disulfide bonds, we tested the impact of the three most commonly manipulated physical parameters; temperature, time and strength of induction. We consistently observed that growth temperatures had the most profound impact on improving protein production in SHuffle cells. This may be due to the fact that SHuffle cells are under oxidative stress, and the resulting detrimental effects may be compounded by high metabolic activity during growth at high temperatures such as 37°C. This hypothesis is supported by the observation that over-expression of poorly folding proteins such as vtPA at 37°C in SHuffle cells is toxic (data not shown).

We observed very efficient production of proteins to high yields when SHuffle cells were grown overnight in Magic Media, reaching final yields of 400 mg/l in the case of a cellulase (with a single disulfide bond). To validate the role of the media, we produced vtPA in Magic Media and observed a 6 fold increase in the final yields compared to standard expression conditions using IPTG as an inducer. This form of protein expression in SHuffle cells may indeed be optimal, even though the mechanism of expression is not clear. Although the exact composition of Magic Media is not disclosed, it is designed to be used for the auto-expression of proteins under the control of the *lac* promoter. The principle of autoexpression is based on diauxic regulation where glucose is the preferred carbon source which results in the repression of the *lac* promoter and upon its consumption, cells switch to growth on lactose which results in the induction of the *lac* promoter [39]. However, β-galactosidase activity is needed to convert lactose to allolactose, the natural inducer of the lactose operon [61]. In the case of the SHuffle B T7 cells, the T7 RNA polymerase gene 1 is inserted into the *lacZ* gene, rendering it inactive. Thus, another mechanism of expression other than autoinduction must be occurring, which is why we termed this form of expression “autoexpression” instead of autoinduction.

In this study, we focused on improving folding of target substrate proteins by manipulating the strain and the conditions of expression. However, for optimal expression of proteins, many other parameters must be manipulated. For example, all proteins which require disulfide bonds for their folding will be secreted to compartments where disulfide bond formation can occur. Thus, they will all have some sort of a signal sequence at their N-terminus. However, to express these proteins in the cytoplasm, a signal sequenceless version of the target protein must be expressed. Removal of the 5’ signal sequence will alter the composition and structure of the mRNA, which is known to play a key role in the expression level of the target protein [62]. One remedy to this potential problem is to fuse the target protein to the carboxyl terminal of MBP, which is known to enhance solubility and can be proteolytically removed post production [49]. Otherwise, using the appropriate expression vector with the optimal promoter, codon usage and ribosome binding site need to be considered for optimal expression of the target protein.

Since bottlenecks in the folding pathway of any given protein are specific to that protein, we explored whether we could increase protein yield by co-expressing various helper proteins. We chose a subset of helper proteins based on either prior experimentation which has shown their utility, or in assumptions based on the helper proteins properties. Redox-active helper proteins had the biggest effect. Co-expression of mutant thioredoxins and PDI homologs were the most successful class of helper proteins. Surprisingly, co-expression of the catalase *katG* improved the activity of vtPA 10-fold. This observation supports the notion that the SHuffle cells are under oxidative stress and boosting the cell’s defenses against oxidative damage can increase the capacity of the cells to produce correctly folded disulfide bonded proteins. However, the decrease in vtPA activity when additional DsbC was expressed from the helper plasmid accentuates the fact that, for each individual protein, there can be an optimum level of a redox helper, with a decrease in activity at amounts higher or lower than that optimum. A similar decrease in activity was observed in the case of periplasmic expression of vtPA [17]. Overexpression of periplasmic DsbC resulted in loss in vtPA activity and eventually resulted loss of viability. The authors attributed the loss in viability to a dramatic reduction in the oxygen uptake rate when DsbC was over-expressed [17]. It is plausible that a similar interaction is occurring in the cytoplasm. This drop in activity was not observed when the putative disulfide bond isomerase from *Aquifex aeolicus* (cAaDsbC) was co-expressed. This difference highlights the protein specificities that govern the interaction between the oxido-reductase and its substrate protein.

Expression of proteins in the cytoplasm instead of in the periplasm is of great advantage. Not only does one avoid the complication of having to secrete the target substrate, the periplasm is devoid of ATP, has only a few ATP-independent chaperones, and is only ~20% of the volume of cytoplasm [63]. The advantage of cytoplasmic expression was observed in the case of vtPA, which had two fold increase in activity when expressed in the cytoplasm [8]. Similarly, we observed a 7 fold increase in the activity of an α1,3 Galactosidase from *Xanthomonas manihotis* having a single disulfide bond, when expressed in the cytoplasm instead of the periplasm (data not shown).

Although cytoplasmic expression may improve the activity of certain proteins, cytoplasmic disulfide bond formation may sometimes be detrimental to certain biological processes. For example, cytoplasmic assembly of the *E. coli* phage M13 appears to be problematic, as SHuffle strains were incapable of forming infective phage (data not shown). In addition, SHuffle cells grown in minimal media under high dissolved oxygen rates showed poor growth when glycerol was the sole carbon source (data not shown). This may be due to altered redox state of SHuffle cells’ metabalome. For example, the *cydAB* operon, which is under the regulation of the ArcAB two component system [64], shows a delayed response in transcriptional activity when shifting from aerobiosis to anerobiosis in SHuffle cells (data not shown). This is most likely due to the silencing of ArcB kinase activity by the oxidation of its cytoplasmic redox-active cysteine residues [65]. These observations highlight our current lack of understanding of the redox biology of SHuffle cells, with many important questions remaining unanswered. How do SHuffle cells cope with oxidizing and reducing conditions within cytoplasm? Which reductases are involved in the oxidation of substrate proteins? What is the role of cytoplasmic oxidized DsbC in disulfide bond formation? How do SHuffle cells perform in high density fermentations? Proteomic and mass spectrometric approaches to address these questions are now in progress.

The SHuffle strains and the expression conditions presented in this report represent the first detailed analysis of the conditions required for efficient cytoplasmic expression and folding of disulfide bonded proteins. The results should allow the expression of previously inaccessible production of proteins in *E. coli*. These SHuffle strains greatly expand the cell biologists toolkit by enabling the use of bacterial production in place of more cumbersome eukaryotic expression systems.

### Conclusions

We have demonstrated the value in engineering an *E. coli trxB gor* suppressor strain which expresses active cytoplasmic DsbC. We found that temperature is of paramount importance and should be optimized for the optimal expression of a substrate protein. Autoexpression of proteins using Magic Media was also very helpful in improving yield. We found several intriguing redox related differences between the *E. coli* B and K12 versions of this strain and showed that the *E. coli* B version of SHuffle strains were superior to its K12 counterpart. Further improvements were made by co-expressing various helper proteins. These SHuffle strains along with the knowledge gained regarding their use will be of great use to the protein expression community.

## Methods

### Bacterial strains, media, and chemicals

Bacterial strains and plasmids were constructed by using standard genetic procedures. List of strains used is summarized in supplementary materials Table 1. SHuffle K12 cells were engineered based on the *trxB gor* suppressor SMG96 [32]. A signal sequenceless *dsbC* construct under the regulation of *rrnB* promoter was integrated into SMG96 using the lambda inch method [66]. SHuffle B strains are based on NEB express cells (C2523) and were constructed using the dithiothreitol (DTT) filter disk method, as described prior [32]. While the commercial names of the SHuffle strains are SHuffle (for the K12 versions) and SHuffle express (for the B versions), we will refer to these strains as SHuffle K12 or SHuffle B for the purposes of clarity. Further versions were engineered by integrating the T7 gene 1 which encodes for the T7 RNA polymerase into *lacZ*, allowing for expression of genes under the regulation of the T7 promoter. A list of plasmids used in this study along with their construction is summarized in supplementary materials Table 2 and 3. Synthetic genes were purchased from Genescript ( http://www.genscript.com). Cells were grown in Rich Media (10 g/L Tryptone, 5 g/L Yeast Extract, 5 g/L NaCl, NaOH to pH 7.2) or in Magic Media (Invitrogen cat# K6803).

### Optimization of protein expression

Three parameters were optimized sequentially in the following order; temperature of growth, time of induction and strength of induction. All experiments were conducted in duplicate samples. Initially, -80°C strain stocks were used to inoculate 5 ml rich media with the appropriate antibiotics (200 μg/ml ampicillin, 40 μg/ml Kanamycin or 10 μg/ml Chloramphenicol). The following day, 25 ml of rich media in 125 ml shaker flask supplemented with antibiotics were inoculated with 250 μl (1/100^th^) of overnights and grown at 30°C for 3 hours until mid-log phase, set as default time of induction for the first step of optimization. The cultures were induced with 1 mM isopropyl-β-D-thiogalactopyranoside (IPTG) set as the default concentration of IPTG and temperature was shifted to 16°C, 25°C, 30°C or 37°C and grown respectively overnight for low temperatures (16°C or 25°C) or another 7 h for higher temperatures (30°C or 37°C). Cells were harvested by centrifugation, lysed by sonication and samples were standardized to equal amounts of protein using Bradford reagent. The optimal temperature of protein expression was determined by measurement of enzymatic activities of crude lysates with appropriate enzymatic tests. The second step of optimization was focused on the time of induction using the optimal temperature from the previous step. Cultures were inoculated as previously described. Cultures were induced either at the time of inoculation (Early induction) or at mid-log phase (Mid induction) or at late-log phase of growth (Late induction). Downstream processes were the same as previously described. Strength of induction was tested by inducing cultures at various IPTG concentrations from 0 mM to 1 mM IPTG. Cells were inoculated as previously described and grown at 30°C until optimal time of induction. Various amount of IPTG were added and cultures were incubated at optimal temperature of protein production. Enzymatic activities were measured from crude lysates as previously described.

### Co-expression of helper proteins

Cultures were grown in rich media supplemented with 0.2% L-arabinose (Sigma Aldrich A3256) to induce co-expression of helper proteins and grown with optimal growth and induction conditions as previously described. Appropriate enzymatic activities were measured from crude lysates using protocol described previously.

### Autoexpression

Cultures were inoculated and grown in Magic Media (Invitrogen cat# K6803) until reaching optimal time of induction. The temperature was shifted to the optimal temperature of production.

### Protein activity assays

#### Urokinase assay

Urokinase activity was quantified using a coupled reaction in a microtiter plate. 50 μl of soluble protein were added to wells containing 50 mM Tris pH 8, 60 mM 6-aminohexanoic acid (Sigma Aldrich, cat# 07260), 0.1 mg/ml Bovine Plasminogen (American Diagnostica, cat# 416) and 0.4 mM Spectrozyme PL (American Diagnostica, cat# 251) to a final volume of 150 μl. The plate was incubated at 37°C and absorbance at 405 nm was measured for 2 or 3 h until reaching plateau. Activity is directly proportional to A_405nm_ at linear range standardized to protein amount at A_595nm_ using Bradford reagent.

#### tPA assay

Plasminogen activation was quantified using a coupled reaction in a microtiter plate. 50 μl of soluble protein were added to wells containing 50 mM Tris–HCl (pH7.4), 0.01% Tween 80, 0.04 mg/ml human glu-plasminogen (American Diagnostica, cat # 400), and 0.4 mM Spectrozyme PL (American Diagnostica, cat # 251), to a 250 μl final volume. The plate was incubated at 37°C and absorbance at 405 nm was measured after 2 or 3 h until reaching plateau. Activity is directly proportional to A_405nm_ at linear range standardized to protein amount at A_595nm_ using Bradford reagent [8].

#### Gluc assay

The Gluc activity was quantified using *Gaussia* Luciferase Assay Kit E3300L (New England Biolabs, cat# E3300).

#### PhoA assay

The PhoA activity was quantified using chromogenic reaction in a clear bottom microtiter plate. 20 μl of soluble protein were added to wells containing 180 μl of 20 mM para-nitrophenyl phosphate (pNPP, Sigma Aldrich, cat # N4645), 1 M Tris pH 8, 1 mM ZnAc. The plate was incubated at 37°C and absorbance at 410 nm was measured for 20 minutes. Activity is directly proportional to A_410nm_ at linear range standardized to protein amount at A_595nm_ using Bradford reagent.

#### AppA assay

AppA activity was quantified as described earlier [12] with slight modifications. Assays were performed in microtiter plates with 20 μl of appropriately diluted soluble protein. Reaction was stopped with 50 μl 5 M NaOH. AppA activity was measured at A_410nm_ and standardized to protein amount at A_595nm_ using Bradford reagent.

#### Chitinase assay

Chitinase activity was quantified by fluorometric assay as follows. In microtiter white opaque plate, a serial dilution (1:4 to 1:256) of 50 μl of soluble protein was added to wells containing 20 mM NaPO4, 200 mM NaCl, 1 mM EDTA, 20uM 4-methylimbelliferyl-N, N’, N”-triacyl-B-chitotrioside (stock in 100% DMSO) (Calbiochem) to 200 μl final volume. The plate was incubated at 25°C and fluorescence (Excitation A_320nm_, Emission A_460nm_) was measured 2 to 3 h. Activity is directly proportional at linear range to A_460nm_ standardized to protein amount at A_595nm_ using Bradford reagent.

#### CelZ assay

Activity was measured by incubation of known quantities of celZ with the chromophore p-nitrophenylcellobioside at 50°C, in 50 mM HEPES, pH 7.2 for 30–60′ in 50μL volumes. Reactions were stopped and color developed by the addition of 12.5μL 10% w/v NaCO3 and read at 410 nm.

#### Cel9A assay

Activity was measured by digests of carboxymethylcellulose (CMC). Reactions were carried out with known quantities of protein in 50μL volumes of 1% w/v CMC (med. viscosity, Fluka) for 30–60′ at 50°C in 50 mM HEPES, pH 7.2. Reducing sugars liberated were measured using the 3,5-dinitrosalysilic acid (DNS) method with a panel of glucose standards, read at 540 nm. Activity is expressed in glucose equivalents.

### Protein purification

#### vtPA and gluc

Cells expressing either His tagged vtPA, or His tagged GLuc from various plasmids were grown with shaking in 500 mL Rich Medium supplemented with appropriate antibiotics. Optimal amount of IPTG was added after optimal time of growth at 30°C and the cultures were grown for an additional time at optimal temperature. Cells were harvested by centrifugation (12000 rpm, 20 min, 4°C) and resuspended in Phosphate Buffer (20 mM Phosphate Buffer, 500 mM NaCl, 20 mM Imidazole), and lysed using sonication 8 × 30s. The insoluble fractions were removed be centrifugation (14000 rpm, 30 min, 4°C). Protein was purified using a HiTrap IMAC FF 1 mL column (GE Healthcare), eluting with 1 M Imidazole. Fractions containing protein are pooled, dialyzed in storage buffer (200 mM NaCl, 20 mM Tris HCl pH 7.5, 1 mM EDTA, 0.1% Triton X-100, 50% Glycerol), and loaded on a SDS-PAGE gel. Protein amount was determined by Bradford assay using BSA as standard. Corresponding assay were performed on the purified samples as described before.

#### Chitinase, AppA and PhoA

Cells expressing His tagged Chitinase, AppA or PhoA from various plasmids were grown and harvested as described before. The pellet was resuspended in Tris binding buffer (20 mM Tris pH 8, 300 mM NaCl, 10 mM Imidazole) and purification was performed as described before.

#### Cellulase purification

Individual colonies were picked in duplicate and used to inoculate 5 mL LB-carb starter cultures at 37°C. Starter cultures were measured for growth by OD_600nm_ and used to inoculate either 50 or 100 mL cultures of Magic Media + 100 μg/mL carbenicillin in 250 or 500 mL (respectively) baffled flasks to a density of 0.05. Cells were grown at 37°C until OD_600nm_ reached 1.0 at which time, temperature was dropped to 22–25°C and cultures were grown for a total of 24 h, and harvested when 2 consecutive OD_600nm_ measurements (taken at 0.5 h intervals) showed no increase in density. Cells were immediately put on ice and transferred to cold 50 mL conical bottom tubes, then centrifuged at 4°C for 30′ at 3500 rpm. Cells were resuspended in 10 mL lysis buffer: 1xPBS (teknova), PMSF, leupeptin, pepstatin, 1 mg/mL lysozyme (egg white, Sigma), 1U/mL DNase I. Pellets were disrupted by sonicating for 5 minutes (30s on, 30s off) on ice. A sample was taken for T. Disrupted cells were spun down at 3500 rpm for 30′ at 4°C. 4 mL fractions of the supernatant were diluted with 2x binding buffer (40 mM imidazole, 1 M NaCl, 0.1 M phosphate, pH 7.5) and centrifuged cold to remove new precipitations. 8 mL volumes were loaded onto a 1 mL HisTrap FF column, washed with 12 Column Volumes (CV) binding buffer, and eluted on a 20–140 mM imidazole gradient, collected in 5 mL fractions (Bio-Rad Biologic LP + BioFrac). Purified proteins were quantitated by the Bradford method (Bio-Rad kit). Specific activity was determined using the corresponding enzymatic assay.

### Protein sample analysis

#### AMS alkylation

Cells were grown in rich media supplemented with antibiotics until reaching late log phase of growth (5 h). OD600nm was measured and cultures were diluted to the lowest OD. 3 samples of 1 ml culture were incubated on ice for at least 15 minutes with 15% trichloroacetic acid (TCA). The supernatant was discarded after centrifuging 10 min at maximum speed. The pellets were washed with 500 μl Acetone, mixed by vortex and centrifuged for 5 min at maximum speed. The pellets were air dried and resuspended in 150 μl of either loading buffer (1X Loading buffer, 1% SDS, 0.1 M Tris pH8), 4-acetamido-4′-maleimidylstilbene-2,2′-disulfonic acid (AMS) buffer (15 mM AMS, 1X Loading buffer, 1% SDS, 0.1 Tris pH8) or DTT buffer (100 mM DTT, 1X Loading buffer, 1% SDS, 0.1 Tris pH8). The samples were boiled for 20 minutes at 95°C and incubated at 4°C overnight. Samples resuspended in DTT buffer were incubated on ice for at least 15 minutes with 15% TCA and centrifuged for 10 minutes at maximum speed. The pellet was washed with 500 μl Acetone and air dried. The pellet was resuspended in AMS buffer. 15 μl of samples was loaded on a SDS-PAGE gel and probed with appropriate antibody.

#### Western blot

Samples were diluted 1:3 in 1x Loading Buffer (New England Biolabs, B7709) supplemented with 1x DTT. Samples were loaded on Daichi pre-cast 10/20 gels (Cosmo Bio Co. LTD, cat# 414893) and run for 1 h at 30 mA per gel. Proteins were transferred on PVDF (IPVH00010 Milipore) membranes using wet transfer methods for 1.5 h at 500 mA. Membrane was blocked with 5% Dry Milk (BioRad, 170-6404XTU) in PBS (Gibco, AM9625) for 1 h at room temperature or overnight at 4°C. Membrane was washed 3 × 5min in PBS, Tween 0.05% and incubated with appropriate antibody diluted in PBS-T Dry Milk 1% for 1 h at room temperature. After washing the membrane as described previously the membrane was incubated with secondary antibody if needed diluted in PBST for 1 h at room temperature. After washing as described above the membrane was poured with 20X LumiGLO® Reagent and 20X Peroxide (#7003 Cell signaling technology) for 30 s. The signal intensity was measured.

## Competing interests

MB and PR are owners of New England Biolabs Stock.

## Authors’ contributions

JL conducted most of the experiments, CE and CJ conducted the cellulase studies, MF constructed the SHuffle B strains, PR helped with the protein purification and MB designed and wrote the manuscript. All authors read and approved the final manuscript.

## Supplementary Material

Additional file 1: Figure 1 Growth of SHuffle and wt *E. coli* at 30°C. Growth of various strains monitored for 30hrs at 30°C. Time point of mid (solid arrow) and late (dotted arrow) induction are shown. **(A)** Growth curves of K12 strains. **(B)** Growth curves of B strains [67,68]. Click here for file
