# Erratum to: SHuffle, a novel *Escherichia coli* protein expression strain capable of correctly folding disulfide bonded proteins in its cytoplasm

**DOI:** 10.1186/s12934-016-0512-9

**Published:** 2016-07-13

**Authors:** Julie Lobstein, Charlie A. Emrich, Chris Jeans, Melinda Faulkner, Paul Riggs, Mehmet Berkmen

**Affiliations:** New England Biolabs, Ipswich, MA 01938 USA; Allopartis Biotechnologies, San Francisco, CA 94158 USA; QB3-MacroLab, University of California, Berkeley, CA 94720 USA; Bradley University, Peoria, IL 61625 USA; New England Biolabs, 240 County Road, Ipswich, MA 01938 USA

## Erratum to: Microbial Cell Factories 2012, 11:56 DOI: 10.1186/1475-2859-11-56

After publication of this work [1], it was noted that supplementary figures were missed within the additional files: additional file 1 contained missing figures. The correct version of Additional file 1 can be found here.

## Additional file

10.1186/s12934-016-0512-9 Growth of SHuffle and wt E. coli at 30 °C. **Figure S2.** Western blot analysis of vtPA indicates correct folding in SHuffle B and not in SHuffle K12. **Figure S3.** Expression of flag tagged helper proteins in SHuffle B cells. **Table S1.** List of strains used in this study. **Table S2.** List of plasmids used in this study. **Table S3.** List of primers and the sequences used in construction of the plasmids.
