# Supplementary material for: SHuffle, a novel Escherichia coli protein expression strain capable of correctly folding disulfide bonded proteins in its cytoplasm
Source: Microb Cell Fact. 2012 May 8;11:56. doi: 10.1186/1475-2859-11-56 (PMC3526497; doi:10.1186/1475-2859-11-56)

**Supplementary Figure 1.** Growth of SHuffle and wt *E. coli* at 30˚C. Growth of various strains monitored for 30hrs at 30˚C. Time point of mid (solid arrow) and late (dotted arrow) induction are shown. (A) Growth curves of K12 strains. (B) Growth curves of B strains.


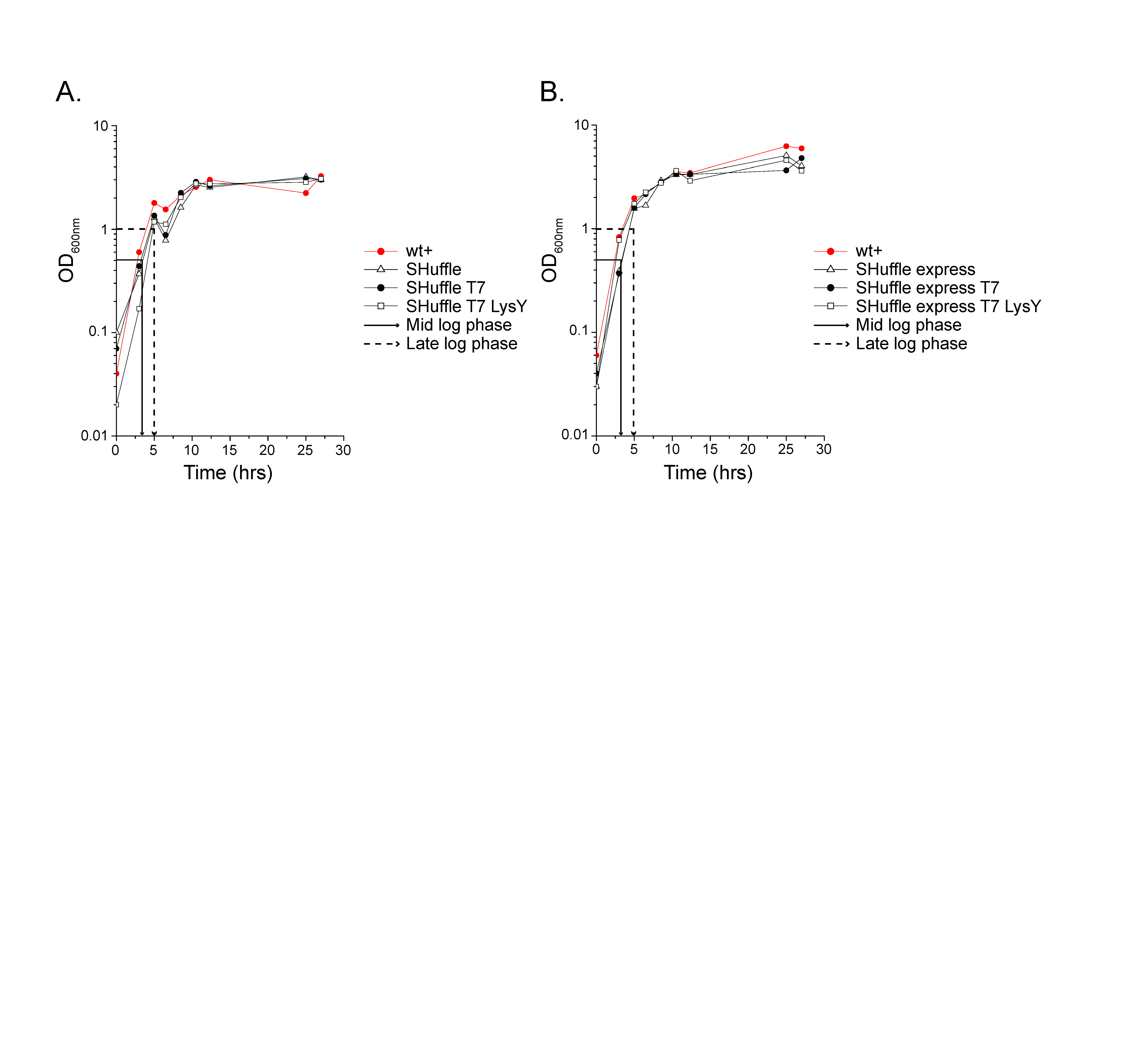

Supplement: Additional file 1: Figure 1 — Growth of SHuffle and wt E. coli at 30°C. Growth of various strains monitored for 30hrs at 30°C. Time point of mid (solid arrow) and late (dotted arrow) induction are shown. (A) Growth curves of K12 strains. (B) Growth curves of B strains [67,68]. [file 1475-2859-11-56-S1.doc]
