# Supplementary material for: Erratum to: SHuffle, a novel Escherichia coli protein expression strain capable of correctly folding disulfide bonded proteins in its cytoplasm
Source: Microb Cell Fact. 2016 Jul 13;15:124. doi: 10.1186/s12934-016-0512-9 (PMC4944262; doi:10.1186/s12934-016-0512-9)
Supplement: Supplementary file 1 — 10.1186/s12934-016-0512-9 Growth of SHuffle and wt E. coli at 30 °C. Figure S2. Western blot analysis of vtPA indicates correct folding in SHuffle B and not in SHuffle K12. Figure S3. Expression of flag tagged helper proteins in SHuffle B cells. Table S1. List of strains used in this study. Table S2. List of plasmids used in this study. Table S3. List of primers and the sequences used in construction of the plasmids. [file 12934_2016_512_MOESM1_ESM.pdf]

Figure S1. Growth of SHuffle and wt *E. coli* at 30˚C. Growth of various strains monitored for 30hrs at 30˚C. Time point of mid (solid arrow) and late (dotted arrow) induction are shown. (A) Growth curves of K12 strains. (B) Growth curves of B strains.

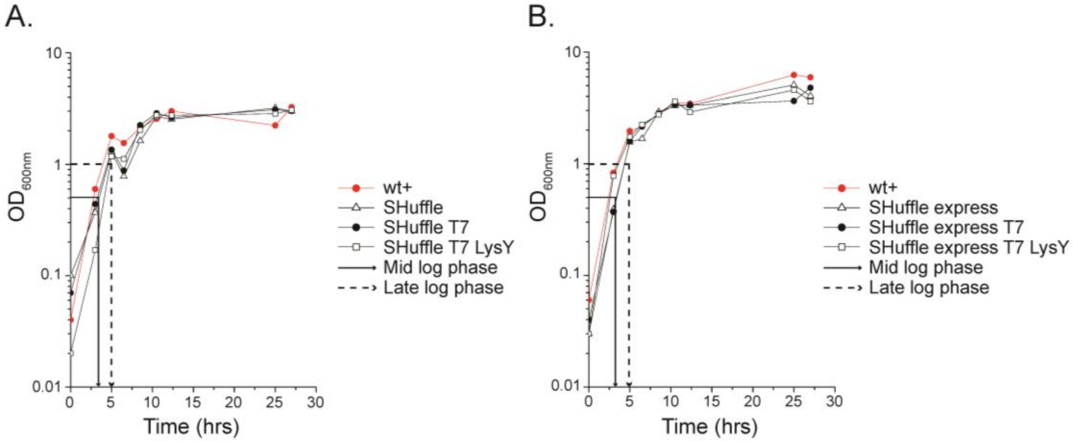

Figure S2. Western blot analysis of vtPA indicates correct folding in SHuffle B and not in SHuffle K12. Cultures were grown in rich media at 30º C for 5 hours followed by induction with 1 mM IPTG at 16º C overnight. Samples were equilibrated according to their OD_{600}, boiled in the presence of 50 mM DTT, separated in SDS-PAGE and transferred to PVDF. The blot was probed with monoclonal anti-HIS antibody (Novagen cat# 70796-3). Lane 1: MB2093, lane 2: MB2285 and lane 3 MB2585. Expected molecular weight of vtPA is 40.3 kD, indicated by an arrow.

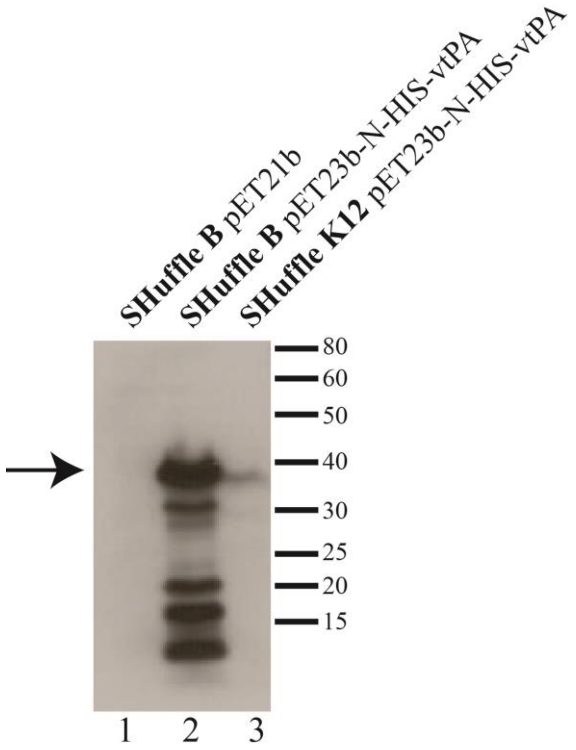

Figure S3. Expression of flag tagged helper proteins in SHuffle B cells. Cultures were grown in rich media at 30º C for 5 hours followed by induction with 0.2% L-arabinose at 16º C overnight. Samples were equilibrated according to their OD_{600}, boiled in the presence of 50 mM DTT, separated in SDS-PAGE and transferred to PVDF. The blot was probed with monoclonal HRP-conjugated anti-flag antibody (Sigma-Aldrich cat# A8592). The two non-specific cross-reacting bands are indicated with red stars. The molecular weight of the markers (M) is shown in kD (A). Full length PDI can be detected when the film is subjected to longer exposure (B).

A

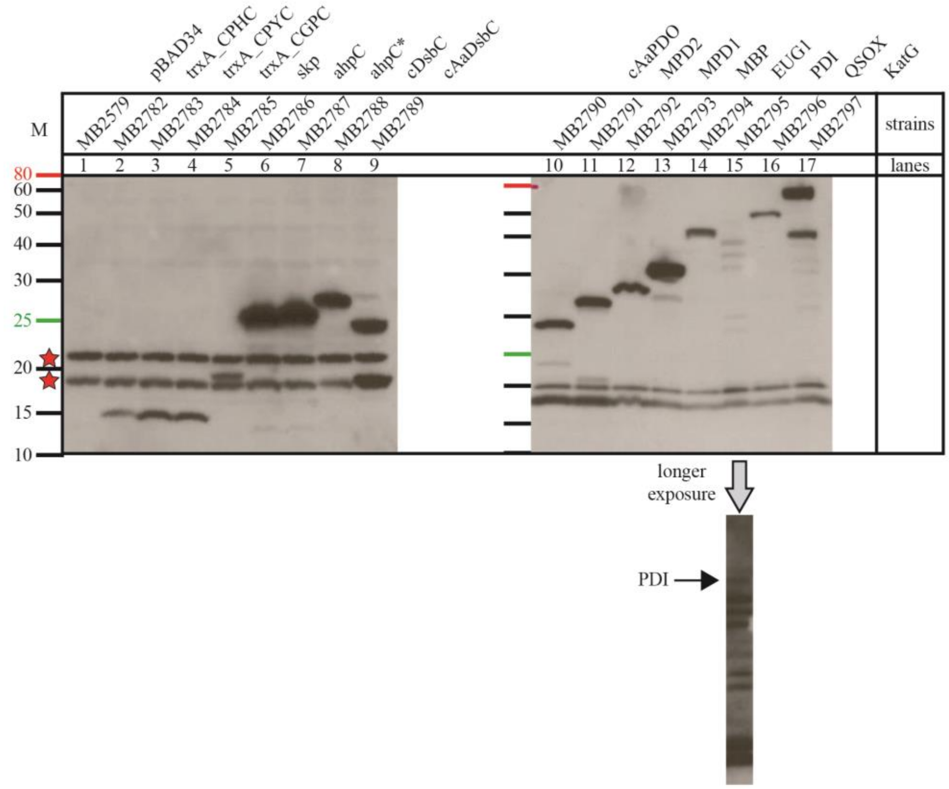

B

Table S1. List of strains used in this study.

Strains	Relevant genotype or features	Reference or source
NEB express T7	*E. coli BL21 fhuA2 lacZ::T7 gene1 [lon] ompT gal sulA11 R(mcr-73::miniTn10*--Tet^{S}*)2 [dcm] R(zgb-210::Tn10-*-Tet^{S}*) endA1* Δ*(mcrC-mrr)114::IS10*	NEB cat# C2566
NEB express	*E. coli BL21 fhuA2 [lon] ompT gal sulA11 R(mcr-73::miniTn10*--Tet^{S}*)2 [dcm] R(zgb-210::Tn10*--Tet^{S}*) endA1* Δ*(mcrC-mrr)114::IS10*	NEB cat# C2523
SHuffle	*E. coli* F´ *lac pro lacI*^{q} */ Δ(ara-leu)7697 araD13 fhuA2 Δ(lac)X74 Δ(phoA)PvuII phoR ahpC* galE (or U) galK Δλatt::pNEB3-r1-cDsbC* (Spec^{R}, *lacI*^{q}) *ΔtrxB rpsL150(Str*^{R}*) Δgor Δ(malF)3*	NEB cat# C3025
SHuffle T7	*E. coli* F´ *lac, pro, lacI*^{Q} */ Δ(ara-leu)7697 araD139 fhuA2 lacZ::T7 gene1 Δ(phoA)PvuII phoR ahpC* galE (or U) galK λatt::pNEB3-r1-cDsbC* (Spec^{R}, *lacI*^{q}) *ΔtrxB rpsL150(Str*^{R}*) Δgor Δ(malF)3*	NEB cat# C3026
SHuffle express	*E. coli BL21 fhuA2 [lon] ompT ahpC gal λatt::pNEB3-r1-cDsbC* (Spec^{R}, *lacI*^{q}) *ΔtrxB sulA11 R(mcr-73::miniTn10*--Tet^{S}*)2 [dcm] R(zgb-210::Tn10* --Tet^{S}*) endA1 Δgor* Δ*(mcrC-mrr)114::IS10*	NEB cat# C3028
SHuffle express T7	*E. coli BL21 fhuA2 lacZ::T7 gene1 [lon] ompT ahpC gal λatt::pNEB3-r1-cDsbC* (Spec^{R}, *lacI*^{q}) *ΔtrxB sulA11 R(mcr-73::miniTn10*--Tet^{S}*)2 [dcm] R(zgb-210::Tn10* --Tet^{S}*) endA1 Δgor* Δ*(mcrC-mrr)114::IS10*	NEB cat# C3029
ER2744	*E. coli fhuA2 lacZ::T7 gene1 glnV44 e14- rfbD1? relA1? endA1 spoT1? thi-1 Δ(mcrC-mrr)114::IS10*	NEB cat# ER2744
DHB4	*F'lac, pro, lacIQ, Δ(malF)3, Δ(phoA)PvuII, phoR, Δ(lac)X174, Δ(ara,leu)7697, araD139, galE(or U), galK*	(67)
SMG96	DHB4 *ΔtrxB, Δgor ahpC**	(32)
MB981	DHB4 pGluc (Amp^{R})	This study
MB986	SMG96 pGluc (Amp^{R})	This study
MB1729	C2523 *ΔtrxB, Δgor ahpC**	This study
MB1731	C2566 *ΔtrxB, Δgor ahpC**	This study
MB1961	C3029 pET24b-ovtPA-HIS (Kan^{R}, low Spec^{R})	This study
MB1976	C3029 pET24b (Kan^{R}, low Spec^{R})	This study
MB2010	C3029 pET11d-PfCHT1 (Amp^{R}, low Spec^{R})	This study

MB2013	C3029 pET24b-urokinase-HIS	(Kan^R, low Spec^R)	This study
MB2036	C3029 pET11d-PfCHT1 + pBAD33	(Amp^R, Cam^R, low Spec^R)	This study
MB2038	C3029 pET11d-PfCHT1 + pBAD33-cAa-*gua*	(Amp^R, Cam^R, low Spec^R)	This study
MB2039	C3029 pET11d-PfCHT1 + pBAD34-cAa-*dsbC*	(Amp^R, Cam^R, low Spec^R)	This study
MB2041	C3029 pET11d-PfCHT1 + pBAD33-*ctrxA*$_{\mathrm{CGPC}}$	(Amp^R, Cam^R, low Spec^R)	This study
MB2042	C3029 pET11d-PfCHT1 + pBAD33-*ctrxA*$_{\mathrm{CPYC}}$	(Amp^R, Cam^R, low Spec^R)	This study
MB2043	C3029 pET11d-PfCHT1 + pBAD33-*ctrxA*$_{\mathrm{CPHC}}$	(Amp^R, Cam^R, low Spec^R)	This study
MB2049	C3029 pET11d-PfCHT1 + pBAD33-*QSOX*	(Amp^R, Cam^R, low Spec^R)	This study
MB2064	C3029 pET24b-urokinase-HIS + pBAD33	(Kan^R, Cam^R, low Spec^R)	This study
MB2066	C3029 pET24b-urokinase-HIS + pBAD33-cAa-*gua*	(Kan^R, Cam^R, low Spec^R)	This study
MB2067	C3029 pET24b-urokinase-HIS + pBAD34-cAa-*dsbC*	(Kan^R, Cam^R, low Spec^R)	This study
MB2069	C3029 pET24b-urokinase-HIS + pBAD33-*ctrxA*$_{CGPC}$	(Kan^R, Cam^R, low Spec^R)	This study
MB2070	C3029 pET24b-urokinase-HIS + pBAD33-*ctrxA*$_{CPYC}$	(Kan^R, Cam^R, low Spec^R)	This study
MB2071	C3029 pET24b-urokinase-HIS + pBAD33-*ctrxA*$_{CPHC}$	(Kan^R, Cam^R, low Spec^R)	This study
MB2077	C3029 pET24b-urokinase-HIS + pBAD33-*QSOX*	(Kan^R, Cam^R, low Spec^R)	This study
MB2090	C3028 pGluc	(Amp^R, low Spec^R)	This study
MB2093	C3029 pET21b	(Amp^R)	This study
MB2150	C2566 *ΔtrxB, Δgor ahpC** Δλatt::pNEB3-r1-c*dsbC* + pET24b-urokinase-HIS	(Kan^R)	This study
MB2151	C2566 *ΔtrxB, Δgor ahpC** Δλatt::pNEB3-r9-c*dsbC* + pET24b-urokinase-HIS	(Kan^R)	This study
MB2152	C2566 *ΔtrxB, Δgor ahpC** Δλatt::pNEB3-r70-c*dsbC* + pET24b-urokinase-HIS	(Kan^R)	This study
MB2259	C3029 pET21b-cIgG-αMBP	(Amp^R, low Spec^R)	This study
MB2285	C3029 pET23b-N-HIS-vtPA	(Amp^R, low Spec^R)	This study
MB2292	C2523 pGluc	(Amp^R)	This study
MB2293	C2523 Δ*trxB,* Δ*gor ahpC** pGluc	(Amp^R)	This study
MB2294	C3025 pCOAT	(Amp^R, low Spec^R)	This study
MB2295	C3025 pGluc	(Amp^R, low Spec^R)	This study
MB2296	C3028 pCOAT	(Amp^R, low Spec^R)	This study
MB2361	C3029 pET23b-cPhoA	(Amp^R, low Spec^R)	This study

MB2362	C3029 pET23b-cAppA	(Amp^R, low Spec^R)	This study
MB2371	C2566 pET24b-urokinase-HIS	(Kan^R)	This study
MB2372	C2566 Δ*trxB,* Δ*gor ahpC** pET24b-urokinase-HIS	(Kan^R)	This study
MB2373	C2566 pET23b-N-HIS-vtPA	(Amp^R)	This study
MB2374	C2566 Δ*trxB,* Δ*gor ahpC** pET23b-N-HIS-vtPA	(Amp^R)	This study
MB2423	C3029 pET11d-PfCHT1 + pBAD33-c*dsbC*	(Amp^R, Cam^R, low Spec^R)	This study
MB2424	C3029 pET11d-PfCHT1 + pBAD34-*malE*	(Amp^R, Cam^R, low Spec^R)	This study
MB2425	C3029 pET11d-PfCHT1 + pBAD34-*PDI*	(Amp^R, Cam^R, low Spec^R)	This study
MB2426	C3029 pET11d-PfCHT1 + pBAD34-*EUG1*	(Amp^R, Cam^R, low Spec^R)	This study
MB2427	C3029 pET11d-PfCHT1 + pBAD34-*MPD1*	(Amp^R, Cam^R, low Spec^R)	This study
MB2428	C3029 pET11d-PfCHT1 + pBAD34-*MPD2*	(Amp^R, Cam^R, low Spec^R)	This study
MB2429	C3029 pET11d-PfCHT1 + pBAD34-*skp*	(Amp^R, Cam^R, low Spec^R)	This study
MB2430	C3029 pET11d-PfCHT1 + pBAD34-*katG*	(Amp^R, Cam^R, low Spec^R)	This study
MB2431	C3029 pET11d-PfCHT1 + pBAD33-*ahpCF*	(Amp^R, Cam^R, low Spec^R)	This study
MB2432	C3029 pET11d-PfCHT1 + pBAD33-*ahpC*F*	(Amp^R, Cam^R, low Spec^R)	This study
MB2333	C3029 pET24b-urokinase-HIS + pBAD33-c*dsbC*	(Kan^R, Cam^R, low Spec^R)	This study
MB2434	C3029 pET24b-urokinase-HIS + pBAD34-MalE	(Kan^R, Cam^R, low Spec^R)	This study
MB2435	C3029 pET24b-urokinase-HIS + pBAD34-PDI	(Kan^R, Cam^R, low Spec^R)	This study
MB2436	C3029 pET24b-urokinase-HIS + pBAD33-EUG1	(Kan^R, Cam^R, low Spec^R)	This study
MB2437	C3029 pET24b-urokinase-HIS + pBAD34-MPD1	(Kan^R, Cam^R, low Spec^R)	This study
MB2438	C3029 pET24b-urokinase-HIS + pBAD34-MPD2	(Kan^R, Cam^R, low Spec^R)	This study
MB2439	C3029 pET24b-urokinase-HIS + pBAD34-*skp*	(Kan^R, Cam^R, low Spec^R)	This study
MB2440	C3029 pET24b-urokinase-HIS + pBAD34-*katG*	(Kan^R, Cam^R, low Spec^R)	This study
MB2441	C3029 pET24b-urokinase-HIS + pBAD33-*ahpCF*	(Kan^R, Cam^R, low Spec^R)	This study
MB2442	C3029 pET24b-urokinase-HIS + pBAD33-*ahpC*F*	(Kan^R, Cam^R, low Spec^R)	This study
MB2460	C3029 pET23b-N-HIS-vtPA + pBAD34	(Amp^R, Cam^R, low Spec^R)	This study
MB2461	C3029 pET23b-N-HIS-vtPA + pBAD33-cAa-*gua*	(Amp^R, Cam^R, low Spec^R)	This study
MB2462	C3029 pET23b-N-HIS-vtPA + pBAD34-cAa-*dsbC*	(Amp^R, Cam^R, low Spec^R)	This study

MB2463	C3029 pET23b-N-HIS-vtPA + pBAD33-*ctrxA*$_{CGPC}$	(Amp^R, Cam^R, low $Spec^R$)	This study
MB2464	C3029 pET23b-N-HIS-vtPA + pBAD33-*ctrxA*$_{CPYC}$	(Amp^R, Cam^R, low $Spec^R$)	This study
MB2465	C3029 pET23b-N-HIS-vtPA + pBAD33-*ctrxA*$_{CPHC}$	(Amp^R, Cam^R, low $Spec^R$)	This study
MB2466	C3029 pET23b-N-HIS-vtPA + pBAD33-QSOX	(Amp^R, Cam^R, low $Spec^R$)	This study
MB2467	C3029 pET23b-N-HIS-vtPA + pBAD33-*cdsbC*	(Amp^R, Cam^R, low $Spec^R$)	This study
MB2468	C3029 pET23b-N-HIS-vtPA + pBAD34-*malE*	(Amp^R, Cam^R, low $Spec^R$)	This study
MB2469	C3029 pET23b-N-HIS-vtPA + pBAD34-PDI	(Amp^R, Cam^R, low $Spec^R$)	This study
MB2470	C3029 pET23b-N-HIS-vtPA + pBAD34-EUG1	(Amp^R, Cam^R, low $Spec^R$)	This study
MB2471	C3029 pET23b-N-HIS-vtPA + pBAD34-MPD1	(Amp^R, Cam^R, low $Spec^R$)	This study
MB2472	C3029 pET23b-N-HIS-vtPA + pBAD34-MPD2	(Amp^R, Cam^R, low $Spec^R$)	This study
MB2473	C3029 pET23b-N-HIS-vtPA + pBAD34-*skp*	(Amp^R, Cam^R, low $Spec^R$)	This study
MB2474	C3029 pET23b-N-HIS-vtPA + pBAD34-*katG*	(Amp^R, Cam^R, low $Spec^R$)	This study
MB2475	C3029 pET23b-N-HIS-vtPA + pBAD33-*ahpCF*	(Amp^R, Cam^R, low $Spec^R$)	This study
MB2476	C3029 pET23b-N-HIS-vtPA + pBAD33-*ahpC*F*	(Amp^R, Cam^R, low $Spec^R$)	This study
MB2579	C3029 pBAD34	(Cam^R)	This study
MB2585	C3026 pET23b-N-HIS-vtPA	(Amp^R)	This study
MB2588	MB1731 Δ*dsbC*::kan	(Kan^R)	This study
MB2782	C3029 pBAD34-TrxA_CPHC-flag	(Cam^R)	This study
MB2783	C3029 pBAD34-TrxA_CPYC-flag	(Cam^R)	This study
MB2784	C3029 pBAD34-TrxA_CGPC-flag	(Cam^R)	This study
MB2785	C3029 pBAD34-skp-flag	(Cam^R)	This study
MB2786	C3029 pBAD34-ahpC-flag	(Cam^R)	This study
MB2787	C3029 pBAD34-ahpC*-flag	(Cam^R)	This study
MB2788	C3029 pBAD34-cDsbC-flag	(Cam^R)	This study
MB2789	C3029 pBAD34-cAaDsbC-flag	(Cam^R)	This study
MB2790	C3029 pBAD34-cAaPDO-flag	(Cam^R)	This study
MB2791	C3029 pBAD34-MPD2-flag	(Cam^R)	This study
MB2792	C3029 pBAD34-MPD1-flag	(Cam^R)	This study

MB2793	C3029 pBAD34-MBP-flag	(Cam^R)	This study
MB2794	C3029 pBAD34-EUG1-flag	(Cam^R)	This study
MB2795	C3029 pBAD34-PDI-flag	(Cam^R)	This study
MB2796	C3029 pBAD34-QSOX-flag	(Cam^R)	This study
MB2797	C3029 pBAD34-KatG-flag	(Cam^R)	This study

Table S2. List of plasmids used in this study.

Plasmids	Source	Template	Primer pair	Restriction Enzyme
pBAD33-cAaPDO	This study	*Aquifex aeolicus* genome	32-34	KpnI/SphI
pBAD34-cAaDsbC	This study	*Aquifex aeolicus* genome	27-26	NcoI/XbaI
pBAD33-TrxA$_{CPHC}$	This study	pDSW204-DsbAss-TrxA	340-276/5	SacI/PstI
pBAD33-QSOX	This study	pHIS-A_HsQSOX1b	45-46	SacI/XbaI
pBAD34-MalE	This study	pMALc5X	126/127	NcoI/XbaI
pBAD34-PDI	This study	pUC57-PDI1 (codon optimized)	Subcloned	NcoI/XbaI
pBAD34-EUG1	This study	pUC57-EUG1 (codon optimized)	Subcloned	NcoI/XbaI
pBAD34-MPD1	This study	pUC57-MPD1 (codon optimized)	Subcloned	NcoI/XbaI
pBAD34-MPD2	This study	pUC57-MPD2 (codon optimized)	Subcloned	NcoI/XbaI
pBAD34-katG	This study	MB1733	158/159	NcoI/XbaI
pET24b-urokinase-HIS	This study	pTrc99a-urokinase	43-44	NdeI/XhoI
pBAD33-TrxA$_{CGPC}$	(68)			
pBAD33-TrxA$_{CPYC}$	(68)			
pBAD33-cDsbC	(8)			
pBAD33-ahpCF	(32)			
pBAD33-ahpC*F	(32)			
pBAD34-skp	Claude Maina			
pGLuc	Ron Chong			
pET11d-PfCHT1	Fana Mersha			
pET23b-cAppA	(29)			
pET23b-cPhoA	(29)			
pET23b-N-HIS-vtPA	(28)			

Table S3. List of primers and the sequences used in construction of the plasmids.

Name	Sequence (5' to 3')
26	ATGCTCTAGATTATTTAACGAGTTCATCAAGC
27	ATGCCATGGCTTCATGCCCTGAACCCTCCAAATTTG
32	ATGCGGTACCATGCTTCTGAACCTGGATGTGAG
34	ATGCGCATGCTTAAGCCTGTTCTTTTTCCCTCTTG
43	ATGCCATATGGGCAGTGTACTTGGAGCTCCTG
44	ATGCCTCGAGGAAGGCCAGACCTTTCTCTTCTCC
45	ATGCGAGCTCAGGAGGACAGCTATGGCTAGCATGACTGGTGGACAGC
46	ATGCTCTAGATCAAATAAGCTCAGGTCCCTCAGCC
126	ATGCCCATGGATGAAAATCGAAGAAGGTAAACTGG
127	ATGCTCTAGATTAAGTCTGCGCGTCTTTCAGGGCTTC
158	ATGCCCATGGGGAGCACGTCAGACGATATCCATAACACC
159	ATGCTCTAGATTACAGCAGGTCGAAACGGTCGAGG
340-275	TATGCCTGCAGTTACGCCAGGTTAGCGTCGAGG
340-276	ATGCGAGCTCATGAGCGATAAAATTATTCACC
